# Integrative Korean medicine treatment for temporomandibular joint disorder-induced dysphagia leading to aspiration pneumonia: A case report (CARE-compliant)

**DOI:** 10.1097/MD.0000000000018013

**Published:** 2019-11-15

**Authors:** Ji Eun Park, Seunghoon Lee

**Affiliations:** Department of Acupuncture and Moxibustion Medicine, College of Korean Medicine, Kyung Hee University, 23 Kyungheedae-ro Dongdaemun-Gu, Seoul, South Korea.

**Keywords:** aspiration pneumonia, case report, dysphagia, integrative Korean medicine treatment, temporomandibular joint disorders

## Abstract

**Introduction:**

Severe temporomandibular joint disorder (TMD) could induce dysphagia, which could lead to aspiration pneumonia. However, no clinical study has reported that TMD-related dysphagia could result in aspiration pneumonia. Integrative Korean medicine (KM) is suggested to be an effective treatment for patients with severe TMD.

**Patient concerns:**

A 76-year-old female could not open her mouth because of TMD and subsequently experienced dysphagia. To clearly identify the cause of dysphagia and to treat the symptoms, she was admitted to the neurology department. However, she eventually developed aspiration pneumonia. Despite the inpatient treatment and even after pneumonia was cured, TMD symptoms and dysphagia persisted.

**Diagnosis:**

Based on the Diagnostic Criteria for Temporomandibular Disorders (DC/TMD) and the magnetic resonance imaging findings, the patient was diagnosed as having severe TMD with disc displacement without reduction and with limited opening.

**Interventions:**

Integrative KM treatment, including acupuncture, herbal acupuncture, cupping therapy, Chuna manual therapy, and herbal medicine, was performed during the admission period (23 days).

**Outcomes:**

The following clinical improvements were detected: maximal unassisted opening from 8 to 28 mm, right lateral movement from 3 to 11 mm, left lateral movement from 10 to 15 mm, and protrusion movement from 5 to 7 mm. Dysphagia disappeared when the TMD symptoms improved.

**Conclusion:**

Patients with severe TMD might experience dysphagia, which could lead to aspiration pneumonia. Symptoms of severe TMD could be treated with integrative KM treatment.

## 1 Introduction

Temporomandibular joint disorder (TMD) includes a wide range of disorders affecting the masticatory musculature, temporomandibular joint (TMJ), and related structures.^[1]^ Based on the National Health Interview Surveys (1989–2009), the prevalence of self-reported TMD symptoms was 5% among adults in the United States.^[2]^ In Korea, the TMD prevalence was 3.1% in 2009.^[3]^ According to the Korean Health Insurance Review and Assessment service, the number of Korean patients with TMD who visited the hospital has increased by ∼ 60% over the past 7 years (2010–2017).^[4,5]^ Moreover, TMD is common in adults aged 20 to 50 years, and it is more prevalent in women than in men.^[6]^

TMD symptoms include pain around the TMJ or temporal region, noise in the TMJ when moving the jaw, decreased mandibular range of motion or jaw deviation when opening the mouth, and impaired oral transit. These symptoms could affect eating, drinking, and swallowing, which in turn could cause oral stage dysphagia (OD). The prevalence of TMD-related OD is reported to be 9.3%.^[7]^ OD could increase the likelihood of oropharyngeal aspiration, which is an important etiologic factor of pneumonia.^[8]^ However, currently, no clinical study has reported that TMD-related dysphagia could result in aspiration pneumonia.

Here, we report a case of a 76-year-old female with severe TMD who developed dysphagia that induced aspiration pneumonia. After receiving integrative Korean medicine (KM) treatment, including acupuncture, herbal acupuncture, cupping therapy, Chuna manual therapy (CMT), and herbal medicine, she could open her mouth and dysphagia was resolved.

## 2 Case report

### 2.1 Clinical features

**Figure 1:**
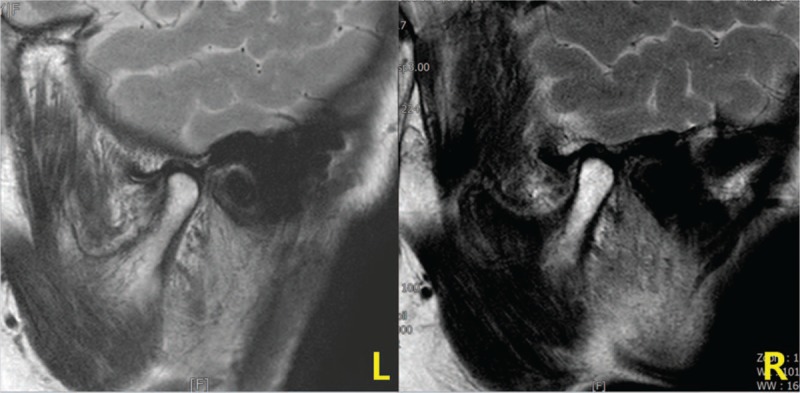
Temporomandibular joint (TMJ) magnetic resonance imaging (MRI). MRI showed anterior displacement of the left TMJ disc and mild anterior displacement of the right TMJ disc (April 17, 2017). L = left, R = right.

**Figure 2:**
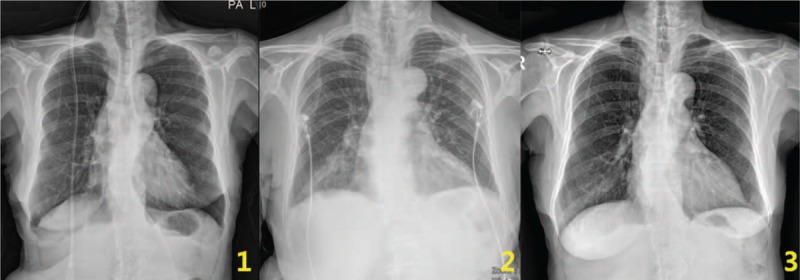
Chest X-ray. (1) No remarkable finding (April 20, 2017). (2) Bronchopneumonia at the right lower lung field (April 23, 2017). (3) No active lung infiltrations (June 7, 2017).

**Figure 3:**
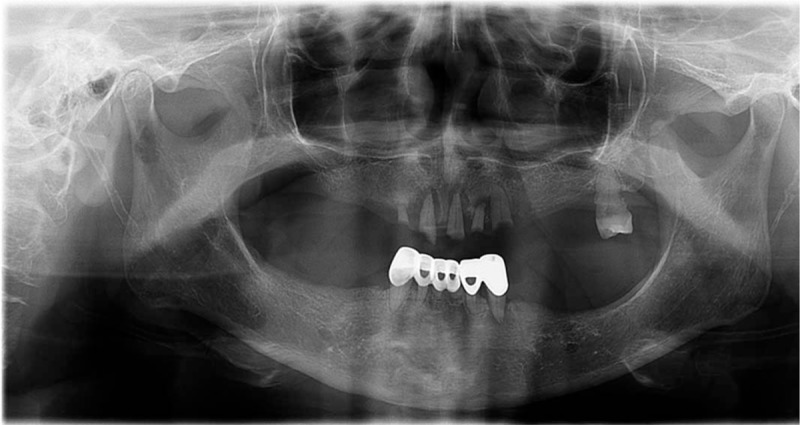
Jaw X-ray. The jaw X-ray showed that the patient had several missing teeth (April 11, 2017).

A 76-year-old Korean female experienced jaw opening restriction after she tried hard to keep her mouth wide open to take out Korean melon pieces that were stuck in her mouth using her fingers. The restriction worsened, she could not open her mouth, and she had trouble swallowing food 7 days after the onset. She visited an emergency center and was diagnosed as having TMD with disc dislocation based on a TMJ X-ray; however, she returned home without any medical treatment. After 10 days since the onset, her symptoms persisted and she was admitted to the hospital at a department of neurology. A nasogastric tube was inserted to prevent aspiration due to dysphagia. To identify the neurological cause of dysphagia, various examinations, such as brain magnetic resonance imaging (MRI) and neck/head computed tomography (CT), were performed; however, the findings were unremarkable. Videofluoroscopic swallow study (VFSS) revealed cricopharyngeal dysfunction, and esophagoscopy showed no definite abnormal lesion. TMJ MRI showed anterior displacement of the left TMJ disc and mild anterior displacement of the right TMJ disc (Fig. 1). Urinalysis and chest X-ray showed no remarkable findings (Fig. 2-1). Blood test also showed no remarkable findings, except blood urea nitrogen (34.9 mg/dL). She did not take any medication and had no family history. She could not wear her denture (Fig. 3) because she could not open her mouth.

Fever and dyspnea occurred 6 days after admission. Inflammatory markers increased (C-reactive protein [CRP] 1.20 mg/dL, white blood cell 12.75 × 10^3^/μL, segmented neutrophil 86.7%) and oxygen saturation decreased (atrial blood gas analysis [ABGA]: pO_2_ 60.4 mm Hg, pCO_2_ 37.7 mm Hg). Chest X-ray suggested bronchopneumonia at the right lower lung field (Fig. 2-2). Group A hemolytic streptococcus was detected with sputum culture, and antibiotics (Tazoperan 4.5 g IV q 8 h) was administered to treat pneumonia. Ten days after admission, CRP further increased (8.14 mg/dL), dyspnea was aggravated (ABGA pO_2_ 43.7 mm Hg), and she required urgent tracheotomy in the intensive care unit. After treatment in the intensive care unit, pneumonia improved; however, she still could not open her mouth. She was discharged from the hospital without proper TMD treatments and opted for integrative KM treatment instead.

When she visited the Korean medical hospital, her maximum unassisted opening (MUO) was 8 mm, left lateral excursion 10 mm, right lateral excursion 3 mm, and protrusion 5 mm. She had pain in the left masticatory muscles (temporalis and master muscle) and around the left TMJ pole when opening her mouth, moving her jaw to the side, and palpating the area. Pain in the left TMJ and temporalis muscle, which was measured by a 100-mm visual analog scale (VAS), was 16.5 and 31 mm, respectively. She was diagnosed as having disc displacement without reduction with limited opening according to the Diagnostic Criteria for Temporomandibular Disorders (DC/TMD): Diagnostic Decision Tree.^[9]^ Her Jaw Functional Limitation Scale-8 (JFLS-8) score was 52 (the scale was translated into Korean by the authors). This report just documented a previous observational data and did not include any sensitive personal information; therefore, ethical approval was not necessary. Written informed consent was obtained from the patient for the publication of this case.

### 2.2 Integrative Korean medicine treatment

The patient received integrative KM treatment, which included acupuncture, herbal acupuncture, cupping therapy, CMT, and herbal medicine, during the admission period (May 16, 2017 to June 7, 2017).

#### 2.2.1 Acupuncture

Acupuncture treatment (10-mm depth) was performed using 0.25 × 40-mm disposable stainless steel needles (DongBang Acupuncture Inc., Boryung, South Korea) once a day for 20 min. The acupuncture points were the following: left GB3, ST7, SI19, ST4, ST6, TE20, GB20, and EX-HN5. A 4-Hz electric stimulation (STN–111; Stratek, Anyang, South Korea) was applied to the left ST4-ST6, ST7-SI19, and TE20-GB20.

#### 2.2.2 Bee venom herbal acupuncture

A single 0.5 cm^3^ dose of 1:30,000 bee venom (BV) was administered using a 1 cm^3^ disposable syringe (30 gauge; Hwajin Medical Co., Seoul, South Korea) in the left TMJ region once a day, after testing negative in the allergic reaction skin test. Dried BV (10 mg; Yoomil Garden, Hwasun, South Korea) was diluted with 300 cm^3^ (1:30,000) of saline (Joongwe Pharmaceuticals, Seoul, South Korea).

#### 2.2.3 Cupping therapy

Dry cupping therapy was performed using Hansol cupping apparatus (Hansol Medical Co., Ltd., Paju, South Korea) on the posterior neck region and upper trapezius muscle at eight tender points once a day for 5 min.

#### 2.2.4 Herbal medicine

The herbal decoctions “Cheongsangbohangtang,” which consisted of Paeoniae Radix Alba, Rehmanniae Radix Preparat, Liriopes Radix, Platycodi Radix, Trichosanthis Semen, Armeniacae Semen, and Pinelliae Rhizoma (May 17–25, 2017), and “Samultang variants,” which consisted of Paeoniae Radix Alba, Ligustici Rhizoma, Angelicae Gigantis Radix, Rehmanniae Radix Preparat, Platycodi Radix, Trichosanthis Semen, and Armeniacae Semen (May 26–June 7, 2017), were administered three times a day.

#### 2.2.5 Chuna manual therapy

CMT is a manipulation technique that includes mobilization within the limit of the passive range of joint motion and muscle relaxation with the patient's breathing. CMT was started at 4 days after hospitalization, when the patient's MUO was >10 mm. The patient received CMT five times per week.

##### 2.2.5.1 Myofascial release technique

The patient was in the supine position and the practitioner stood beside the affected side of the patient. Circular friction and compression using the second or fifth finger was performed (5–15 s) on the lateral side of the maxillary molar region where insertion of the lateral pterygoid muscle is located.

##### 2.2.5.2 Manipulation technique

With the patient in the supine position, the practitioner grabbed patient's jaw with a thumb placed on the molar and pulled the patient's jaw downward and forward 2 to 3 times to provide a joint space for the placement of TMJ disc.

### 2.3 Clinical outcomes

The MUO, lateral and protrusive movements, pain VAS, and JFLS-8 were evaluated. MUO increased from 8 to 28 mm. Right lateral movement increased from 3 to 11 mm. Left lateral movement increased from 10 to 15 mm. Protrusion movement also increased from 5 to 7 mm (Table 1). The pain VAS of the left TMJ and temporalis muscle decreased from 16.5 to 3 mm and 31 to 10 mm, respectively. JFLS-8 score decreased from 52 to 20.

**Table 1:**
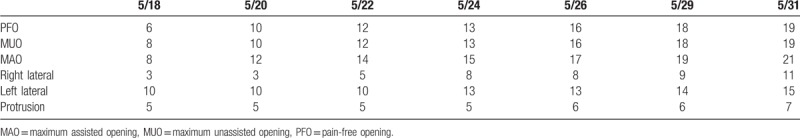
Change in the range of movement of the temporomandibular joint (mm).

**Figure 4:**
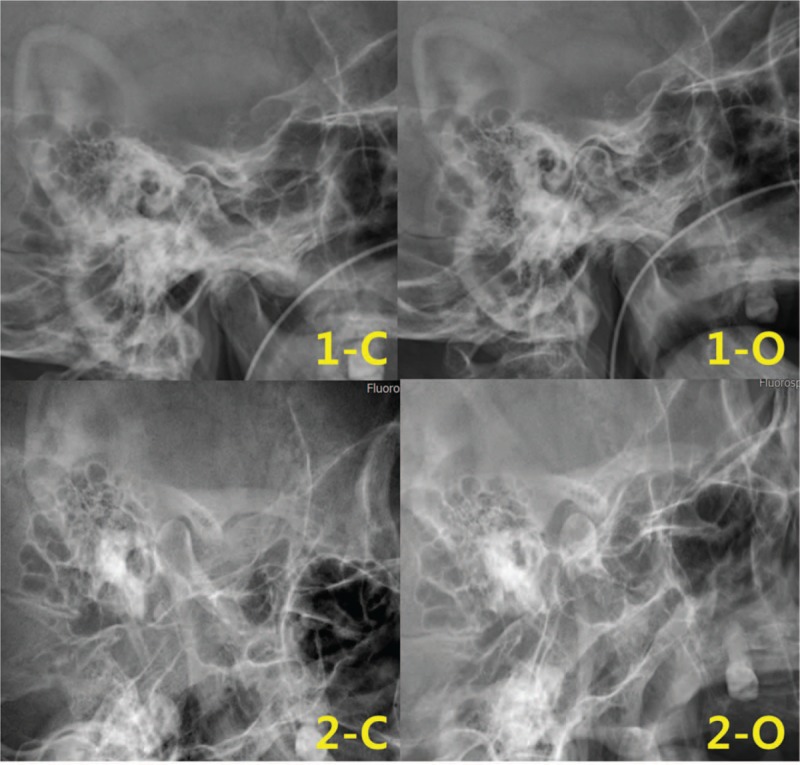
Left temporomandibular joint X-ray. (1) The left mandibular condyle remained in the mandibular fossa even when she attempted to open her mouth widely (May 17, 2017). (2) The left mandibular condyle was slightly translated to the mandibular fossa and moved forward to the articular tubercle (June 7, 2017). C = close, O = open.

Based on the left TMJ X-ray taken on the day of admission, the patient's mandibular condyle remained in the mandibular fossa even when she attempted to open her mouth widely (Fig. 4-1). Twenty-two days after admission, TMJ X-ray showed that the left mandibular condyle was slightly translated to the mandibular fossa and moved forward to the articular tubercle (Fig. 4-2).

**Figure 5:**
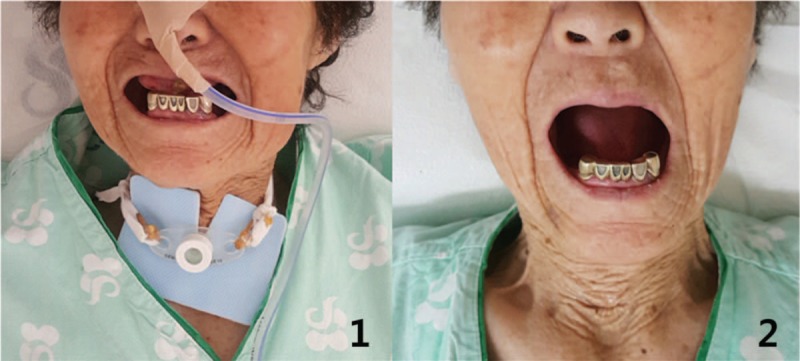
Maximum unassisted opening. (1) The patient's maximum unassisted opening was 8 mm. She was having a tracheotomy tube and a Levin tube (May 16, 2017). (2) The patient's maximum unassisted opening was 28 mm. The tracheotomy and Levin tubes were removed (June 7, 2017).

**Figure 6:**
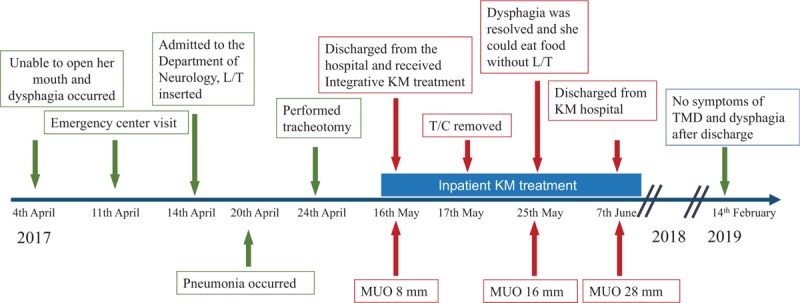
Timeline of the case. KM = Korean medicine, L/T = Levin tube, T/C = tracheotomy, TMD = temporomandibular disorder, MUO = maximum unassisted opening.

The tracheotomy tube was removed by an otorhinolaryngologist 1 day after admission, and the patient could breathe voluntarily. She could eat soft and liquid diet without aspiration when she could open her mouth at 15 mm and after removing the Leven tube 9 days after admission (Fig. 5). She was scheduled for VFSS to evaluate her swallowing function; however, she did not undergo the test because she could swallow without any problems before the scheduled evaluation. Chest X-ray was obtained on the day of discharge and no evidence of pneumonia was found (Fig. 2-3). She was followed up by telephone on February 14, 2019, and we confirmed the absence of TMD symptoms and dysphagia after discharge (Fig. 6).

No severe adverse effect was observed during the treatment period. Minor bleeding at the acupuncture site or BV injection site was observed. During the admission period, one of her front teeth, which had been shaking in the past, was missing regardless of the treatment.

### 2.4 Patient perspective

“I suddenly could not open my mouth, and I could not eat anymore. I later developed pneumonia and I thought I was going to die. When I went to the hospital, the doctor prescribed many tests, but I could not receive any treatment and my symptoms did not get better. My son searched the Internet and brought up hopes that the KM Hospital could treat me. I was treated with integrative KM and was able to open my mouth, and I thought that I could still live. I have been doing well without recurrence for 2 years now after I was discharged from the KM Hospital.”

## 3 Discussion

In this study, we report a case of severe TMD that resulted in difficulty of swallowing, which led to aspiration pneumonia. The patient had jaw locking in closed position after she opened her mouth wide, and she gradually experienced dysphagia, which eventually induced aspiration pneumonia. The patient was diagnosed as having disc displacement without reduction with limited opening. The symptoms of severe TMD were treated with integrative KM treatment.

The etiology of TMD is complex and multifactorial. The possible predisposing factors to TMD could be genetic, hormonal, and anatomical in origin, factors that may precipitate it include trauma, occlusal changes, and parafunction, and factors that may prolong it are stress and parafunction.^[10]^

Prolonged mouth opening (hyperextension), which could lead to microtrauma to the TMJ and subsequent muscular hyperactivity, is also suggested as a possible cause of TMD.^[11]^ Extensive mouth opening could result in increased TMJ muscle tension. A rapid increase in TMJ muscle tension adversely affects the functioning of the TMJ, and an extremely high load often results in significant surface and disc damage within the joints,^[12]^ anterior disc displacement, and a closed lock.^[13]^

TMD-related functional difficulties could impair eating, drinking, and swallowing, thereby causing OD. The mechanism by which TMD directly causes dysphagia is unknown; however, for the case in this study, the following mechanism may be considered: First, jaw movement restriction possibly causes a reduction in oral preparatory mechanisms and results in OD signs and symptoms, such as increased oral transit times and reduced cohesive bolus formation, which could be attributed to masticatory difficulties, pain, and fatigue. Second, the lack of posterior teeth also causes reduction in intraoral proprioception mechanism and vertical dimension of the oral cavity, which possibly predisposed the patient to swallowing difficulties. Lack of teeth increases the likelihood of TMD. Although the prevalence is reduced in older people, TMD occurs in 40% of the people who wear a denture.^[14]^ Lastly, orofacial muscle pain leads the patient with TMD to perform compensatory motor adjustments for swallowing, including premature escape and the persistence of food residues in the oral cavity, which could in turn alter tongue position thereby leading to chocking during mastication and deglutition.^[15]^

Integrative KM treatment was used to treat TMD, which consisted of acupuncture, BV herbal acupuncture, cupping therapy, CMT, and herbal medicine. Increased MUO and pain relief in this study are consistent with the results of a study conducted by Cue et al,^[16]^ who reported that acupuncture may produce microstretch effects on the adjacent shortened muscle fibers by evoking small local twitches. Several studies have reported the effects of acupuncture on TMD and suggested that acupuncture improves pain in TMD^[17]^ and could improve mouth opening and jaw movement.^[18]^ We selected the acupuncture points that coincide with the TMJ and its related structures (i.e., lateral pterygoid, masseter, and temporalis muscle) and could help increase mouth opening, improve jaw function, and relieve pain. Herbal acupuncture in the TMJ could relieve TMD-related pain, and one trial^[19]^ reported that it was more effective than analgesic agents. In addition, 14-day topical skin application of BV in TMD patients has more myorelaxant and analgesic effect than placebo.^[20]^

Moreover, CMT is a specialized type of manual therapy in Korea where the practitioner uses manual and/or physical force with optional devices to apply appropriate correcting force to specific body areas to treat various dysfunctions and pathophysiologic condition.^[21]^ We performed CMT to provide a TMJ space for disc reduction. CMT could equalize the length of the sarcomeres in the involved MTrPs,^[22]^ causing reactive hyperemia in that region or a spinal reflex mechanism that relieves muscle spasm.^[23]^ CMT showed functional recovery and treatment effects similar to those of acupuncture in adult patients with TMD and was more effective when combined with acupuncture than when performed alone.^[24]^

Cupping on the posterior neck region could relieve pain and tension in the posterior neck muscles by stimulating the inhibitory receptive fields of cortically projecting multireceptive dorsal horn neurons.^[25]^ The improvement in TMD symptoms with cupping on the neck could be attributed to the biomechanical and neuroanatomical relationships between the neck and jaw.^[26]^

The herbal formula “Cheongsangbohangtang” was prescribed to improve the patient's pneumonia. After the symptoms have improved, we prescribed “Samultang variants,” which consisted of *Paeoniae Radix Alba* as a major compound, to improve blood deficiency syndrome diagnosed by traditional KM theory. *Paeoniae Radix Alba* is effective for muscle pain and cramp,^[27]^ acts directly on the skeletal muscle, and induces relaxation without deterioration of the central nervous system function.^[28]^ Thus, it is presumed to be effective in TMD treatment by reducing the burden on the joint disc though muscle relaxation, and it may have a synergistic effect resulting in positive clinical outcomes.

Hence, the integrative KM treatment improved the MUO, right and left lateral movements, and protrusive movement and relieved the pain of the patient. The patient's JFLS-8 score suggested that the overall functional status of the masticatory system improved. The movement of the muscles involved in swallowing was restored, and the dysphagia was also resolved.

This case report has a limitation. Because the patient received a combination therapy, we could not determine the efficacy of the individual interventions included in the integrative KM treatment. While recent clinical practice guidelines published in Korea^[29]^ recommend acupuncture treatment for TMD patients with moderate level of evidence, further high-quality investigations for BV herbal acupuncture, herbal medicine, and CMT are needed to identify the exact effect of each treatment.

In conclusion, patients with severe TMD may experience dysphagia and, if not treated properly, it could result in aspiration pneumonia. In addition, given the promising results in our case, integrative KM could be a valuable treatment option for patients with severe TMD. Nevertheless, further rigorous randomized studies with a long-term follow-up period and large sample size are warranted to confirm the effectiveness and safety of integrative KM treatment for severe TMD.

## Acknowledgments

We would like to thank Dr Yejin Hong for her assistance with integrative Korean Medicine treatment.

## Author contributions

**Conceptualization:** Seunghoon Lee.

**Funding acquisition:** Seunghoon Lee.

**Investigation:** Seunghoon Lee.

**Methodology:** Seunghoon Lee.

**Project administration:** Seunghoon Lee.

**Writing – original draft:** Ji Eun Park, Seunghoon Lee.

**Writing – review & editing:** Seunghoon Lee.

Seunghoon Lee: 0000-0003-0088-2296.

Seunghoon Lee orcid: 0000-0003-0088-2296.
